# Probiotic Modulation of Gut Microbiota Enhances Immunity and Nutrition in SIT *Ceratitis Capitata* Sterile Males

**DOI:** 10.1007/s00248-026-02754-x

**Published:** 2026-04-13

**Authors:** Kamel Charaabi, Haytham Hamden, Salma Fadhel, Nesrine Tanfouri, Sana Bouzenbila, Wafa Djobbi, Ameur Cherif, Meriem Msaad Guerfali

**Affiliations:** 1 Laboratory of Biotechnology and Nuclear Technologies, LR16CNSTN01, National Centre of Nuclear Sciences and Technologies, Sidi Thabet, Tunisia; 2 ISBST, BVBGR-LR11ES31, University of Manouba, Biotechpole Sidi Thabet, Ariana, Tunisia

**Keywords:** *Ceratitis capitata*, Gut microbiome modulation, Probiotic consortium (L.E.K-PC), Immunocompetence, Metabolic reserves, Sterile insect technique (SIT)

## Abstract

The gut microbiome significantly influences insect physiology, immunity, and ecological fitness. However, mass-rearing and sterilization processes in Sterile Insect Technique (SIT) programs disrupt this natural balance. The resulting dysbiosis is a critical factor reducing the performance of sterile males. This study demonstrates that strategically administered probiotic supplementation can restore gut homeostasis and enhance the nutritional status and immunity of *Ceratitis capitata* sterile males. Our selected three-strain probiotic consortium L.E.K-PC (*Lactococcus lactis*,* Klebsiella oxytoca*, and *Enterobacter* sp.) was administered during, larval (L+), adult (A+) and both life stages (AL⁺), followed by a challenge with the entomopathogenic bacteria *Pseudomonas* sp. Full-length 16 S rRNA Nanopore sequencing revealed that AL⁺ males maintained a diverse and balanced gut microbiota with elevated levels of beneficial bacteria, whereas control flies exhibited marked dysbiosis. Probiotic supplementation significantly increased protein, carbohydrate, and lipid reserves in both life stages, upregulated the expression of antimicrobial peptide genes (*Cecropin*,* Attacin* and *PGRP*), enhanced phenoloxidases activity, and strengthened the antibactericidal capacity of hemolymph. Consequently, AL⁺ males showed the highest survival rate following infection. These results indicate that probiotic supplementation acts as “immunobiotic” coordinating nutritional support and immune activation through gut microbiota modulation. Incorporating such probiotic strategies into SIT programs could enhance sterile male resilience and pathogen resistance, offering a sustainable approachto optimizing mass-rearing and strengthening ecological pest management.

**Supplementary Information:**

The online version contains supplementary material available at 10.1007/s00248-026-02754-x.

## Introduction

The Mediterranean fruit fly, *Ceratitis capitata*, is a highly invasive and economically devastating agricultural pest worldwide. It infests a broad range of fruit and vegetable crops, causing significant global yield losses [1, 2]. Native to sub-Saharan Africa, this species has spread extensively globally through trade and human-mediated transport, establing populations across most tropical and subtropical regions, as well as in temperate zones such as the Mediterranean basin, Central and South America, parts of North America, and Australia [3, 4]. This pest is highly polyphagous, with over 300 recorded host plant species, and shows a strong preference for soft, fleshy fruits such as citrus, stone fruits and pome fruits, as well as several vegetable crops, including tomato and pepper [5]. Its high reproductive rate, broad host range, and remarkable ecological adaptability make ita particularly challenging pest to manage across diverse agroecosystems [3].

The SIT is a non-chemical, environmentally sustainable pest management strategy with strong potential for the long-term control of *C. capitata*, addressing both economic and ecological concerns. Unlike conventional methods that rely heavily on insecticide applications, which often lead to environmental contamination and resistance evolution. SIT is based on the mass rearing, sterilization, and periodic release of sterile males. These males’ mate with wild females, producing infertile offspring and thereby progressively suppressing the target population [6–8]. The success of SIT hinges critically on the physiological quality of the released sterile males. To compete effectively with wild males, they must exhibit high longevity, robust immune competence, and strong mating competitiveness, traits essential for successful field performance and sustained population suppression [9, 10]. However, mass-rearing practices, artificial diets, and sterilization protocols can substantially compromise males quality by inducing gut dysbiosis, characterized by a loss of microbial diversity and functional capacity [11]. This disruption impairs immune function, disturbs metabolic regulation, and increases susceptibility to opportunistic pathogens [12–14]. Consequently, once released, sterile males face multiple environmental stressors, such as temperature fluctuations, dehydration, nutritional scarcity, and exposure to entomopathogens. These challenges further reduce post-release survival and diminish mating competitiveness [14, 15], ultimately undermining the efficacy of SIT programs.

Within sterile insect technique production, the use of probiotics to restore and modulate gut microbiota has generated significant research interest. A balanced gut microbiota plays a central role in host physiology, enhancing nutrient assimilation, metabolic homeostasis, immune stimulation, and stress tolerance [16]. Probiotic supplementation also holds promise for improving the cost-effectiveness of SIT programs, as it is low-cost, easily scalable, and may mitigate losses linked to suboptimal sterile male performance. though formal economic analyses are still needed. Studies on invasive insects, including *C. capitata*, reveal considerable plasticity in the gut microbiome, with composition varying across geographic regions, host diets, and environmental conditions [11, 17–19]. This adaptability suggests that locally influenced microbial communities may contribute to the species’ ecological success and invasiveness. Despite this variability, members of the family Enterobacteriaceae are consistently identified as dominant components of *C. capitata* gut microbiota. Research has shown that incorporating *Klebsiella oxytoca* into the larval or adult diet of medfly for a single generation significantly enhances several key performance traits of sterile males, including improved mating competitiveness, shorter mating latency, increased longevity under starvation, better flight ability, and accelerated immature-stages development [11, 20, 21]. Similarly, enriching larval or adult diets with *Enterobacter* sp. improves a wide range of fitness-related parameters, such as pupal and adult yield, pupal weight, developmental rate, stress tolerance, flight capacity, and mating success [12]. Both *Klebsiella* and *Enterobacter* species are dominant members of the medfly gut microbiota and positively influence host digestion. Notably, their pectinolytic activity aids in breaking down fruit derived sugars, while their diazotrophic capacity contributes to nitrogen fixation, thereby enhancing the host’s nutritional status [22]. Building on these findings our seminal research [23, 24] employed a targeted screening workflow, specifically tailored to the SIT production protocols, to select a three-strain probiotic consortium (*K. oxytoca*, *Enterobacter* sp., and *Lactococcus lactis*, termed L.E.K-PC), from the gut microbiota of a wild Tunisian *C. capitata* population. Integration of this consortium into larval and/or adult diets over 20 generations of mass-rearing helped restore the diversity and function of the gut microbiota following irradiation-induced disruption. This intervention significantly improved the biological quality of sterile males, enhancing their mating competitiveness, longevity, flight ability, and stress tolerance. In this context, the use of probiotics as “immunobiotics” emerges as a promisingd strategy to modulate immune responses by restoring gut microbial balance and strengthening host defense mechanisms against pathogens.

Infection of *C. capitata* by entomopathogens, such as the entomopathogenic fungus *Purpureocillium lilacinum* [25] and pathogenic bacteria such as *Providencia rettgeri* [26], various *Pseudomonas* species [22, 27–29] and *Serratia marcescens* [30], triggers a rapid and multifaceted innate immune response. This defense is primarily coordinated through two key mechanisms: humoral and cellular immunity. The humoral response involves the activation of immune signaling pathways, namely Toll, Imd, and Jak/STAT, which upregulate the expression of antimicrobial peptides (AMPs) such as defensins, cecropins, and attacins that neutralize invading pathogens [31]. Concurrently, the cellular immune response mobilizes hemocytes to execute phagocytosis, encapsulate pathogens, and drive melanization through the phenoloxidase (PO) cascade [32, 33]. The efficacy of these immune defenses varies with pathogen type and infection severity. However, certain entomopathogens have evolved strategies to suppress host immune signaling or exploit oxidative stress, thereby enhancing their virulence. Concequently, the capacity of sterile males to resist pathogen attack in the field depends on a critical balance between robust immune system functionality and the prevailing environmental microbial pressure.

Previous studies have shown that probiotic supplementation enhances immune function in *C. capitata* by modulating gut microbiota composition and restoring immune homeostasis. For instance, diets enriched with *Enterobacter* sp. Upregulate the expression of *Attacin*, which enhances resistance to bacterial infections, and mitigates irradiation-induced oxidative stress [34]. Moreover, supplementing larval and/or adult diets with the L.E.K-PC probiotic consortium significantly boosts predicted immune-related functions. This was supported by predictive metagenomic analyses of 16 S rRNA data, which revealed functional pathways associated with immunity and host resilience [24].

In this study, we evaluated the impact of an artificially induced *Pseudomonas* sp. infection delivered by injection, on *C. capitata*, analyzing changes in gut microbiota structure and diversity. These analyses were conducted both before and after infection, and in conjunction with supplementation of the L.E.K-PC probiotic consortium administered through larval and/or adult diets. Microbiota composition was assessed using full length 16 S rRNA gene sequencing (V1–V9 region) via Oxford Nanopore Technologies (ONT), which provided high taxonomic resolution and enabled the detection of infection-induced microbial dysbiosis. Furthermore, qRT-PCR was used to quantify the expression of selected immune-related genes in response to probiotic treatment and infection. We further measured phenoloxidase (PO) activity and the antimicrobial properties of the hemolymph, alongside to larval nutritional reserves and adult nutritional indices. These parameters served as indirect indicators of immune modulation and physiological status, reflecting the host’s ability to cope with pathogenic stress. This study provides a comprehensive assessment of how probiotic supplementation modulates gut microbiota, immunity, and physiological resilience in *C. capitata* sterile male under entomopathogenic challenge. The findings offer foundational insights to support microbiome-based enhancements in mass-rearing systems, representing a novel and sustainable strategy to improve biological pest control.

## Materials and Methods

### Insect Rearing on L.E.K Probiotic Consortium-Enriched Diets

The L.E.K. probiotic consortium (L.E.K-PC) was composed of three bacterial strains *Lactococcus lactis* (KY807048), *Klebsiella oxytoca* (KY810531), and *Enterobacter* sp. (KY810513), originally isolated from the gut microbiota of *C. capitata*. These strains were selected based on a probiotic screening process for their potential application in the context of the SIT [23]. Each strain was individually cultured in Luria–Bertani (LB) broth at 37 °C until reaching mid-log phase. Bacterial concentrations were adjusted to 10⁹ CFU/ml by serial dilution and plating. Equal volumes of the three cultures were then combined to prepare the final L.E.K-PC inoculum [24].

A colony of the V8-GSS strain of the Mediterranean fruit fly was maintained for over 20 generations on either probiotic-enriched or control diets. Larvae were reared on a standard Tanaka artificial diet composed of 28% wheat bran, 14% sugar, 7% Torula yeast, 1% HCl (37%), 0.2% sodium benzoate, and 50% water. Four experimental groups were established: control (C) reared on probiotic-free larval and adult diets; (L+) a group receiving the L.E.K-PC (10⁹ CFU/g) only in the larval diet; (A+) a group receiving L.E.K-PC (10⁹ CFU/ml) only in the adult liquid diet (sugar, hydrolyzed yeast, and water at a ratio of 3:1:40); and (AL+) a group receiving supplementation at both adult and larval stages. Larvae were maintained at 23 °C and 80% relative humidity, while pupae and adults were kept at 25 °C and 65% relative humidity. Sterile males for subsequent assays were obtained by irradiating pupae at a dose of 110 Gy two days prior to adult emergence [24].

### Analysis of the Nutritional Reserves in the Hemolymph of *C. Capitata* Larvae and Adults

Given that nutritional reserves may underline differences in physiological traits such as immunity, we investigated key nutritional indices in the hemolymph of third-instar larvae that were collected from each experimental group. This developmental stage exhibits maximal feeding activity and nutrient accumulation, which critically determine adult size and fitness [35]. The body content of protein, carbohydrate, and lipid in individual larvae was quantified using Bradford, Anthrone and Vanillin reagents, respectively, as described by Yuval et al., (1998) [36].

To measure nutritional indices such as triacylglycerides (TAG), proteins, and glucose, hemolymph was collected from *C. capitata* sterile male adults of each experimental group. Prior to collection, each fly was rinsed with sterile water to remove fecal and food particles anesthetized on ice, and subjected to thoracic puncture for hemolymph extraction. The hemolymph was immediately mixed with 0.2% Phenylthiourea (PTU) to inhibit melanization and coagulation. Protein concentration was determined using a BCA Protein Assay Kit (Bio-Basic), with absorbance measured at 562 nm on a microplate reader (IRE96, SFRI). Glucose levels were quantified with a Glucose GO Assay Kit (Sigma-Aldrich) based on glucose oxidase enzymatic activity, absorbance was read at 540 nm. Triacylglyceride content was assayed using a Triacylglyceride Quantification Kit (Sigma-Aldrich), in which TAGs where enzymatically converted to free fatty acids and glycerol, followed by absorbance measurement at 570 nm. For all assays, standard curves were prepared concurrently, and sample concentrations were calculated based on sample volumes and standard reference solutions.

### Infection with *Pseudomonas* sp. Survival Assay

To assess the immune competence of sterile *C. capitata* males, four-days-old adults from each experimental group were challenged via intrathoracic injection with an entomopathogenic bacterium *Pseudomonas* sp [22]. Flies of this age were selected because they are sexually mature, correspond to the typical release age for sterile males in the field applications, and are standard for SIT quality control assays [37]. The bacterial isolate was obtained from the intestine of the *C. capitata* V8-GSS strain using Citrimide agar, a selective medium that inhibits most bacteria except *Pseudomonas* sp. due to its resistance to cetyltrimethylammonium bromide. Following homogenization and incubation at 30 °C for 24–48 h, green-pigmented pyocyanin-producing colonies were selected and cultured in LB broth at 37 °C for 24 h to stationary phase. On the day of infection, the bacterial culture was diluted to an OD₆₀₀ = 1.0, and each four-days-old sterile male was injected intrathoracically with 1 µl of this suspension using a Hamilton syringe. This proceedure generated four experimental infected groups: infected control (C_inf), infected adults from the larval supplemented group (L+_inf), infected adults from the adult supplemented group (L+_inf), and infected adults from the group supplemented at both stages (AL+_inf). A separate non-infected control group was injected with the same volume (1 L) of sterile (1X) PBS. Following infection, all flies were maintained in individual cages with access to water and a sugar-yeast diet (3:1 ratio). Mortality was recorded daily until all individuals in the cohort has died. The experiment included three independent replicates, with 10 flies per replicate.

### Assessment of Phenoloxidase and Antibacterial Activities

The phenoloxidase (PO) and antibacterial activity were measured in the hemolymph collected from live adult flies 24 h post-challenged with *Pseudomonas* sp., following the standard collection procedure without phenylthiourea (PTU) to preserve enzymatic activity. This time point was selected to target an early phase of immune activation in dipterans, during which phenoloxidase activity and antibacterial responses are typically upregulated following bacterial challenge [38, 39].

Phenoloxidase activity was measured according to the method of Hassan et al., (2020) [40]. Briefly, for each sample, 8 µl of hemolymph was diluted in 400 µl of ice-cold PBS (pH 7.4) and centrifuged at 8000 g for 5 min at 4 °C to remove hemocytes. The resulting supernatant was used immediately. A 100 µl aliquot of the supernatant was mixed with 100 µl of 10 mM L-Dopa substrate. After 20 min of incubation at 25 °C, the absorbance of the mixture was measured at 490 nm using a microplate reader. The protein concentration of the hemolymph was determined using a BCA Protein Assay Kit (Bio-Basic). The experiment was performed in three independent replicates, each using hemolymph pooled from multiple adults within the same treatment group for each replicate.

The antibacterial activity of hemolymph against *Pseudomonas* sp. from *C. capitata* adults was assessed using a bacterial growth inhibition assay in sterile 96-well plates. In each well, 40 µL of hemolymph was mixed with an overnight bacterial culture. Control wells contained the bacterial culture mixed with sterile PBS (1X) (negative control), or with (1 mg/mL) tetracycline (positive antibiotic control). Plates were incubated at 30 °C for 12 h, after which bacterial growth was quantified by measuring the absorbance at 600 nm [41]. The assay was performed in triplicate using hemolymph from different adults of the same infected group.

### Analysis of Immune Gene Expression by qRT-PCR

Total RNA was extracted from pools of three whole sterile adult males by biological replicate, collected 24 h post-challenged with *Pseudomonas* sp. using TRIzol™ reagent (Invitrogen) according to the manufacturer’s instructions. Three biological replicates were processed for each of the foor dietary experimental groups (C, L+, A+, AL+),. RNA concentration and purity were assessed using a NanoDrop^®^ spectrophotometer (Thermo Scientific), followed by genomic DNA removal with the RQ1 RNase-Free DNase kit (Promega). The RNA was subsequently purified with the NucleoSpin RNA Clean-up kit (Macherey-Nagel). First-strand cDNA was synthesized from 1 µg of total RNA using the iScript™ cDNA Synthesis Kit (Bio-Rad).

The expression the three immune-related genes *Cecropin*, *PGRP*, and *Attacin*, was quantified through quantitative reverse transcription PCR (qRT-PCR) with *gapdh* and *g6pdh* serving as reference genes (Table S1). Reactions were performed in a CFX Connect™ Real-Time PCR System (Bio-Rad) using SYBR^®^ Green Master Mix (Bio-Rad). The cycling conditions consisted of an initial denaturation at 95 °C for 3 min, followed by 40 cycles at 95 °C for 10 s, 57 °C for 30 s, and 68 °C for 30 s. A melt-curve analysis was performed to confirm amplification specificity.

### DNA Extraction and 16 S rDNA Nanopore Sequencing

The assess the effect of the “L.E.K-PC” consortium on the gut microbiota, *C. capitata* sterile males were dissected 24 h post-infection with *Pseudomonas* sp. For each sample, 15 intestines were dissected from the crop to the hindgut, excluding the Malpighian tubules, were pooled. Three such biological replicates wer analyzed per experimental group (C, L+, A+, AL+). Total genomic DNA was extracted using the One-4-All Genomic DNA Mini-Preps Kit (BioBasic) according to the manufacturer’s instructions. The concentration and purity of the extracted DNA were assessed using a NanoDropTM 2000 spectrophotometer (Thermo Scientific) by measuring the absorbance at 260 nm.

The full-length 16 S rRNA gene amplicon library was prepared from 10 ng of extracted DNA per sample using the 16 S-24-V14 Barcoding Kit (SQK-16S114.24, Oxford Nanopore Technologies, UK). The hypervariable regions V1-V9 were amplified via PCR using barcoded versions of the forward primer 27 F (5′-ATCGCCTACCGTGAC–Barcode–AGAGTTTGATCMTGGCTCAG–3′) and reverse primer 1492R (5′-ATCGCCTACCGTGAC–Barcode–CGGTTACCTTGTTACGACTT–3′). Each 50µL reaction contained 25 µL of Hot Start Taq 2X Master Mix. The PCR conditions included an initial denaturation at 95 °C for 30 s, 25 cycles of 95 °C for 20 s, 55 °C for 30 s, 65 °C for 2 min, with a final extension at 65 °C for 5 min. Post-amplification reactions were treated with 4 µL of EDTA and incubated at room temperature for 5 min. Amplicon concentration and quality for each sample were then quantified using the Qubit dsDNA HS Assay Kit and Qubit 4.0 Fluorometer (Thermo Fisher Scientific, Oregon, USA).

Barcoded samples were pooled equimolarly and purified using Agencourt AMPure XP magnetic beads (0.6× ratio). The purified library was washed twice with 80% ethanol, and eluted in 15 µL of elution buffer. The final 16 S rRNA (V1–V9) amplicon library was quantified by Qubit fluorometry, normalized to 50 fmol, and prepared for sequencing by adding Rapid Adapter, sequencing buffer, and library beads. It was then loaded onto a primed and quality-checked MinION R10.4.1 flow cell (FLO-MIN114, Oxford Nanopore Technologies). Sequencing was performed on a MinION MK1B device (Oxford Nanopore Technologies) for 72 h.

Basecalling of the raw FAST5 data generated over the 72-hour run was performed with Dorado (v7.3.11) within MinKNOW software. Reads were subsequently demultiplexed and converted to FASTQ format using Guppy (v6.3.7). Following, these reads were quality-filtered (Q-score ≥ 8; length 1200 and 1800 bp. Filtered reads were analyzed using the EPI2ME 16 S workflow, with taxonomic assignment performed by BLAST against the NCBI RefSeq database. (www.ncbi.nlm.nih.gov).

### Statistical Analysis

All statistical analyses were performed using R software (version 4.2.0) and GraphPad Prism 8.0.2. Data were first assessed for normality and homogeneity of variances using the Shapiro–Wilk test. Group differences were analyzed using one-way ANOVA followed by Tukey’s HSD post-hoc test when parametric assumptions were satisfied, otherwise, the Kruskal-Wallis test was applied, followed by Dunn’s post-hoc comparisons with Benjamini–Hochberg correction. Survival data following *Pseudomonas* sp. infection were analyzed using Kaplan-Meier survival curves, and differences between treatments were evaluated using the log-rank (Mantel–Cox) test.

For gut microbiota analyses, alpha diversity indices (Observed OTUs, Chao1, Shannon, and Simpson) were calculated using the phyloseq and vegan packages. Differences in alpha diversity among groups were assessed using ANOVA or Kruskal-Wallis tests, based on data distribution. Beta diversity was evaluated using Bray-Curtis dissimilarity matrices (Hellinger-transformed), and community structure differences were visualized through non-metric multidimensional scaling (NMDS) and principal coordinate analysis (PCoA) with the vegan and phyloseq packages. The statistical significance of community differences was tested using PERMANOVA with 999 permutations (vegan package). Differential abundance analysis of bacterial taxa before and after infection was conducted using DESeq2, which models count data using a negative binomial distribution and applies shrinkage estimation of log₂ fold changes. P-values were adjusted for multiple testing using the Benjamini–Hochberg false discovery rate (FDR) correction, and taxa with adjusted *P* < 0.05 were considered significantly differentially abundant. Volcano plots were generated using ggplot2.

Gene expression data obtained by qRT-PCR were analyzed using the 2⁻ΔΔCt method, normalized against two reference genes (gapdh and g6pdh). Relative expression levels were log₂-transformed prior to statistical testing to meet normality assumptions. Differences in gene expression were evaluated using one-way ANOVA followed by Tukey’s post hoc test.

All results are presented as mean ± standard deviation (SD) unless otherwise stated, and statistical significance was set at *P* < 0.05.

## Results

### L.E.K-PC Probiotic Supplementation Enhances Nutritional Reserves in *C. Capitata* Larvae and Adults Hemolymph

**Fig. 1:**
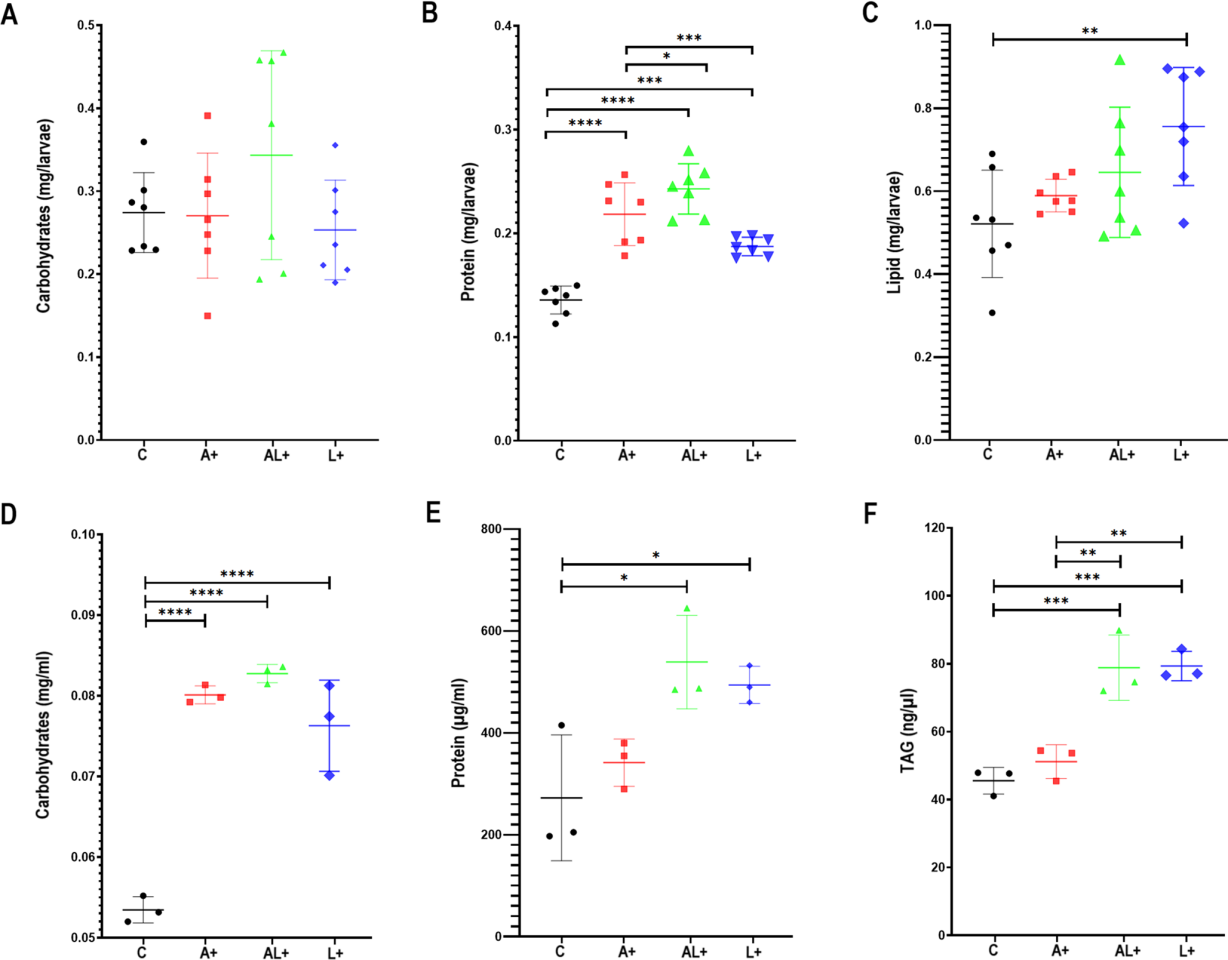
Nutritonal indices in larvae and adult hemolymph of *C. capitata* L.E.K-PC enriched groups. Larval nutritional reserves of carbohydrates (**A**), proteins (**B**), and lipids (**C**), and adult hemolymph content in carbohydrates (**D**), proteins (**E**), and TAG (**F**) in control and L.E.K-PC enriched groups. Data are presented as mean ± SD. Statistical significance was determined by one-way ANOVA followed by Tukey’s post hoc test (*P < 0.05, **P < 0.01, ***P < 0.001, ****P < 0.0001)

Dietary supplementation with the L.E.K-PC significantly affected nutritional physiology in both larvae and adult hemolymph of *C. capitata* sterile males (Fig. 1). In third-instar larvae, total carbohydrate content did not differ significantly among groups (F_3,24_ = 2.99; *P* = 0.051) (Fig. 1A). In contrast, larval protein reserves were significantly increased in all L.E.K-PC treated groups compared to the control, with the highest concentrations detected in the AL⁺ group (0.243 ± 0.024 mg/larvae) (F_3,24_ = 33.79; *P* < 0.0001) (Fig. 1B). A similar effect was seen on larval lipid reserves, which were significantly higher in the supplemented groups, particularly in the L⁺ (0.756 ± 0.142 mg/larvae) and AL⁺ (0.645 ± 0.157 mg/larvae) groups compared to the control (0.521 ± 0.129 mg/larvae) (F_3,24_ = 4.371; *P* < 0.01) (Fig. 1C).

This probiotic-driven enrichment of nutritional reserves was maintained in the adult stage, as reflected in hemolymph composition. Hemolymph carbohydrate (glucose) concentrations were significantly higher in adults from the A⁺ (0.08 ± 0.001 mg/ml) and AL⁺ (0.083 ± 0.001 mg/ml) groups compared to controls (0.053 ± 0.002 mg/ml) (F_3,8_ = 58; *P* < 0.0001) (Fig. 1D). Similarly, total hemolymph protein levels were significantly higher in all L.E.K-PC treated males (F_3,8_ = 6.978; *P* < 0.05), with AL⁺ adults displaying the highest concentrations (539.167 ± 91.663 µg/ml) (Fig. 1E). Triacylglyceride (TAG) levels followed this trend, showing a significant increase in all L.E.K-PC enriched groups (F_3,8_ = 25.47; *P* < 0.001), with the strongest accumulation observed in AL+ (78.838 ± 9.603 ng/µl) and L+ (79.167 ± 91.3.23 ng/µl) males (Fig. 1F).

### Probiotic Supplementation Enhances Immunity and Survival Following Bacterial Challenge

**Fig. 2:**
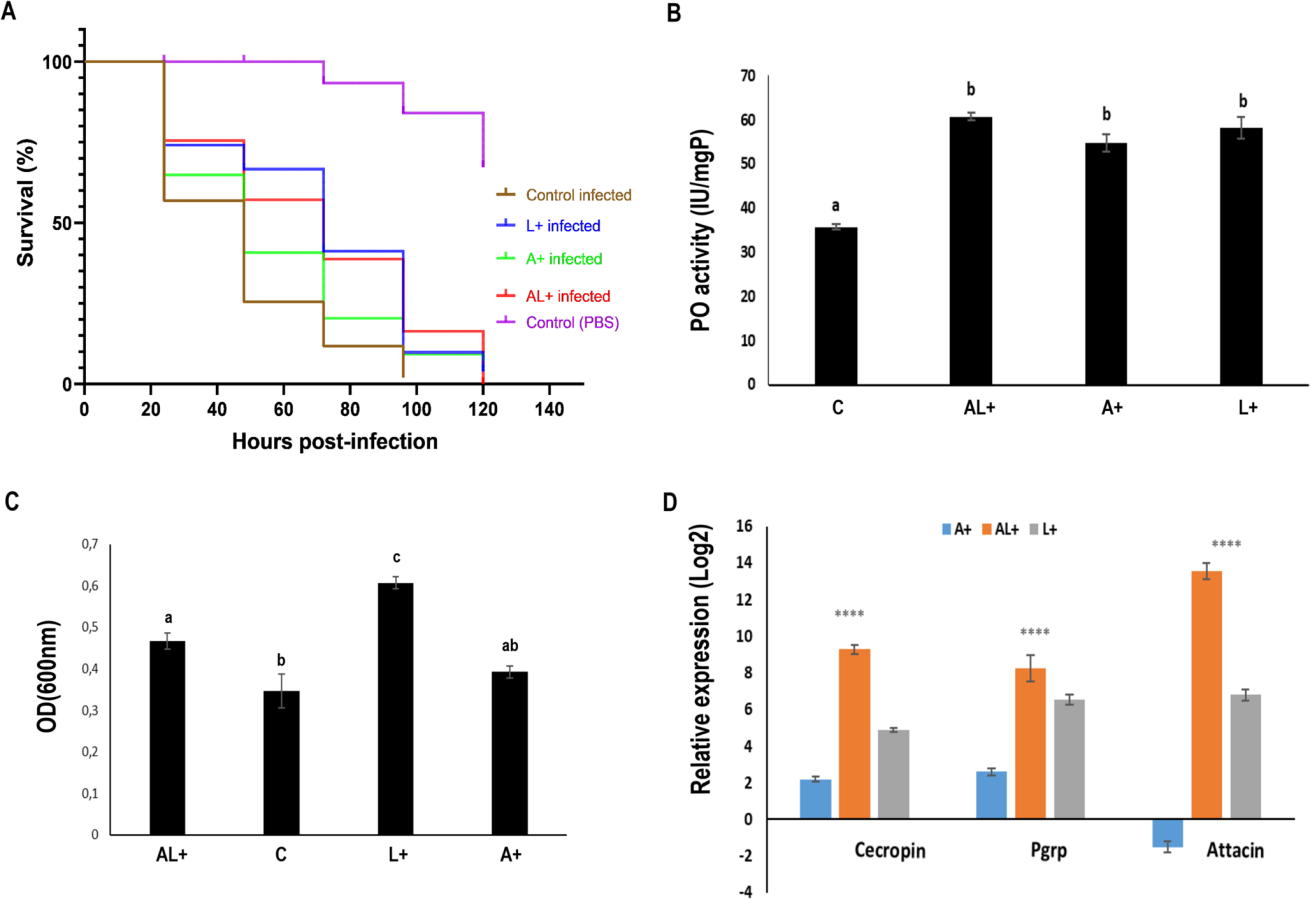
Immune competence and survival of L.E.K-PC fed *C. capitata* males after *Pseudomonas* sp. infection. (**A**) Kaplan–Meier survival curves of different dietary groups after infection (**B**) Phenoloxidase (PO) activity in hemolymph (**C**) In vitro antibacterial activity in hemolymph against *Pseudomonas* sp. (**D**) Relative expression levels of immune-related genes (*Cecropin*,* PGRP*,* Attacin*) analyzed using one-way ANOVA followed by Tukey’s HSD post hoc test. In panel B-D bars represent means ± SD. Different lowercase letters indicate statistically significant differences among treatment groups (*P* < 0.05), while asterisks denote significant differences relative to the control (*P < 0.05, **P < 0.01, ***P < 0.001, **P < 0.0001)

Following *Pseudomonas* sp. infection, L.E.K-PC treatment significantly improved the survival and immune performance of sterile *C. capitata* males (Fig. 2). Survival analysis (Fig. 2A) revealed that all probiotic-fed groups survived longer than the infected controls (χ^2^ = 56.52; df = 4; *P* < 0.0001), with the group supplemented at both life stages AL+ showing the greatest survival over time.

This improved survival was underpinned by a marked enhancement of both cellular and humoral immune functions. Phenoloxidase (PO) activity, a key component of the melanization cascade, was significantly increased in the hemolymph of all L.E.K-PC-treated groups compared to controls (F_3,8_ = 44.35; *P* < 0.0001) (Fig. 2B), with the highest activity observed in AL⁺ males (60.78 ± 0.842 UI/mgP).

Concurrently, antibacterial activity of the hemolymph, assessed against *Pseudomonas* sp., differed significantly among treatments (Fig. 2C). Hemolymph from L.E.K-PC enriched males showed enhanced antibacterial capacity compared to controls (F_3,8_ = 20.55; *P* < 0.0001), with the highest activity observed in the L+ group (0.6 ± 0.014), followed by AL+ (0.46 ± 0.02). These results indicate that L.E.K-PC enrichment, particularly during larval development, strengthens hemolymph-mediated antibacterial defenses in sterile males.

At the transcriptional level, this fortified immune phenotype was associated with a robust upregulation of key immune antimicrobial genes. Quantitative analysis demonstrated that expression of levels of *Cecropin*, *PGRP*, and *Attacin* were significantly higher compared to control group (H = 10.38; df = 4; *P* < 0.0001), particularly in the AL⁺ group (Fig. 2D), indicating robust activation of innate immune pathways.

### The L.E.K-PC consortium modulates gut microbiota structure and diversity under infectious challenge

Long-read sequencing of the V1-V9 hypervariable region of the bacterial 16 S rRNA gene from gut samples yielded a total of 1.262.569 high quality reads, with an average of 52.607 reads per sample and a median length of 1458 bp. The high-quality sequences were classified into 299 operational taxonomic units (OTUs; 97% identity), representing independent species belonging to 49 genera, 20 families, 10 orders, 5 classes across three predominant phyla.

**Fig. 3:**
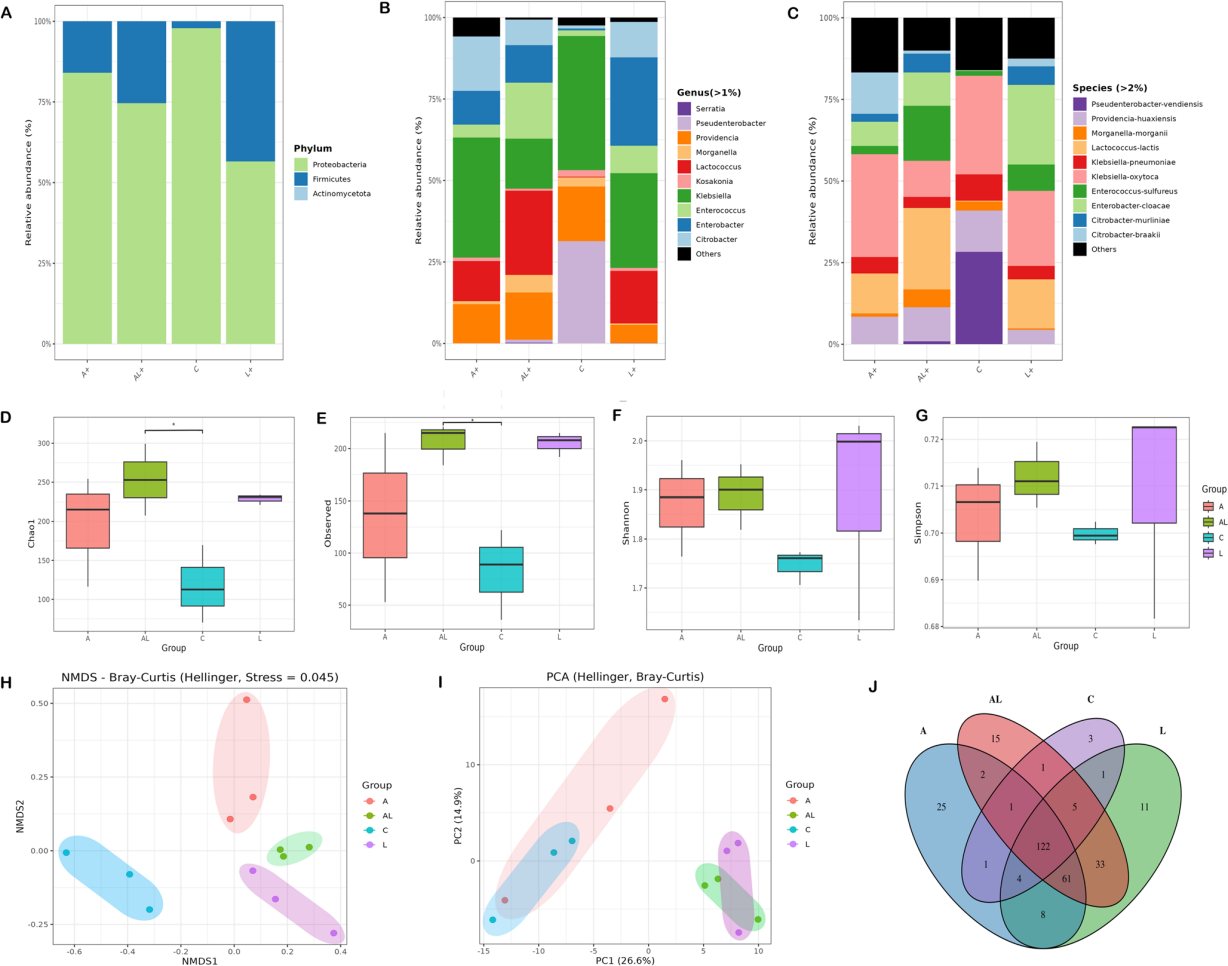
Gut microbiota composition and diversity in *Ceratitis capitata* fed L.E.K-PC diets. Stacked bars show relative abundances of bacteria at the phylum (**A**), genus (**B**), and species (**C**) levels. Box-plots display Alpha-diversity indices (**D-G**). Community dissimilarity (Beta-diversity) is visualized by NMDS (**H**) and PCoA (**I**) plots. Panel J is a Venn diagram of OTU distribution. For panel D-G, bars show means ± SD, and (*) denote statistically significant differences (one-way ANOVA, Tukey’s HSD post hoc test, *P < 0.05, **P < 0.01, ***P < 0.001, ****P < 0.0001))

Significant differences were observed in the taxonomic analysis of the bacterial communities of the gut microbiota of different *C. capitata* groups (C, A+, L+, and AL+) from the phylum to the species level (Table S2). At the phylum level, Pseudomonodota was predominant (F _(3,8)_ = 4.1; *P* < 0.05) but its prevalence varied. While it was predominant in the C, A + and L+ groups, its relative abundance in the AL+ group was nearly equal to that of Bacillota (F_(3,8)_ = 4.1; *P* < 0.05) (Fig. 3A).

At the genus level, *Klebsiella* was the most prevalent in the C (41.19 ± 6.65%), A+ (36.8 ± 8.85%), and AL+ (29.08 ± 4.11%) groups, while *Lactococcus* predominated in the gut microbiota of the L+ group (25.87 ± 9.5%) (Fig. 3B). The dominant microbial species were *Klebsiella oxytoca* in C (30.17 ± 4.32%) and A+ (31.5 ± 8.12%) groups, *Enterobacter cloacae* in the AL+ group (24.36 ± 3.49%) and *Lactococcus lactis* in L+ group (24.92 ± 9.19%) (Fig. 3C) (Table S2).

To compare the gut microbial composition between *C. capitata* groups, the alpha diversity and beta diversity of the microbial community were analyzed. Alpha diversity revealed that the richness was specifically higher in the AL+ group compared to C group as indicated by the Chao1 (F _(3, 8)_ = 4.33; *P* < 0.05) (Fig. 3D) and Observed OTUs values (F _(3, 8)_ = 4.18; *P* < 0.05) (Fig. 3E). However, no significant differences were observed in the Shannon diversity index (F _(3, 8)_ = 0.88; *P* = 0.491) (Fig. 3F) or the Simpson diversity index (F _(3, 8)_ = 2.53; *P* = 0.468) (Fig. 3G). Beta diversity assessments using NMDS (Fig. 3H) and PCoA (Fig. 3I) based on Bray-Curtis distance (Hellinger transformation) revealed distinct clustering of the gut microbiota among the dietary groups (PERMANOVA, *P* = 0.001). This indicates that L.E.K-PC feeding induces notable shifts in microbial community structure. A Venn diagram illustrates the overlap of gut bacterial taxa among the four groups. While 122 core taxa were shared across all treatments, each group also harbored unique taxa, reflecting both a conserved microbial foundation and stage-specific modifications induced by the probiotic (Fig. 3J).

**Fig. 4:**
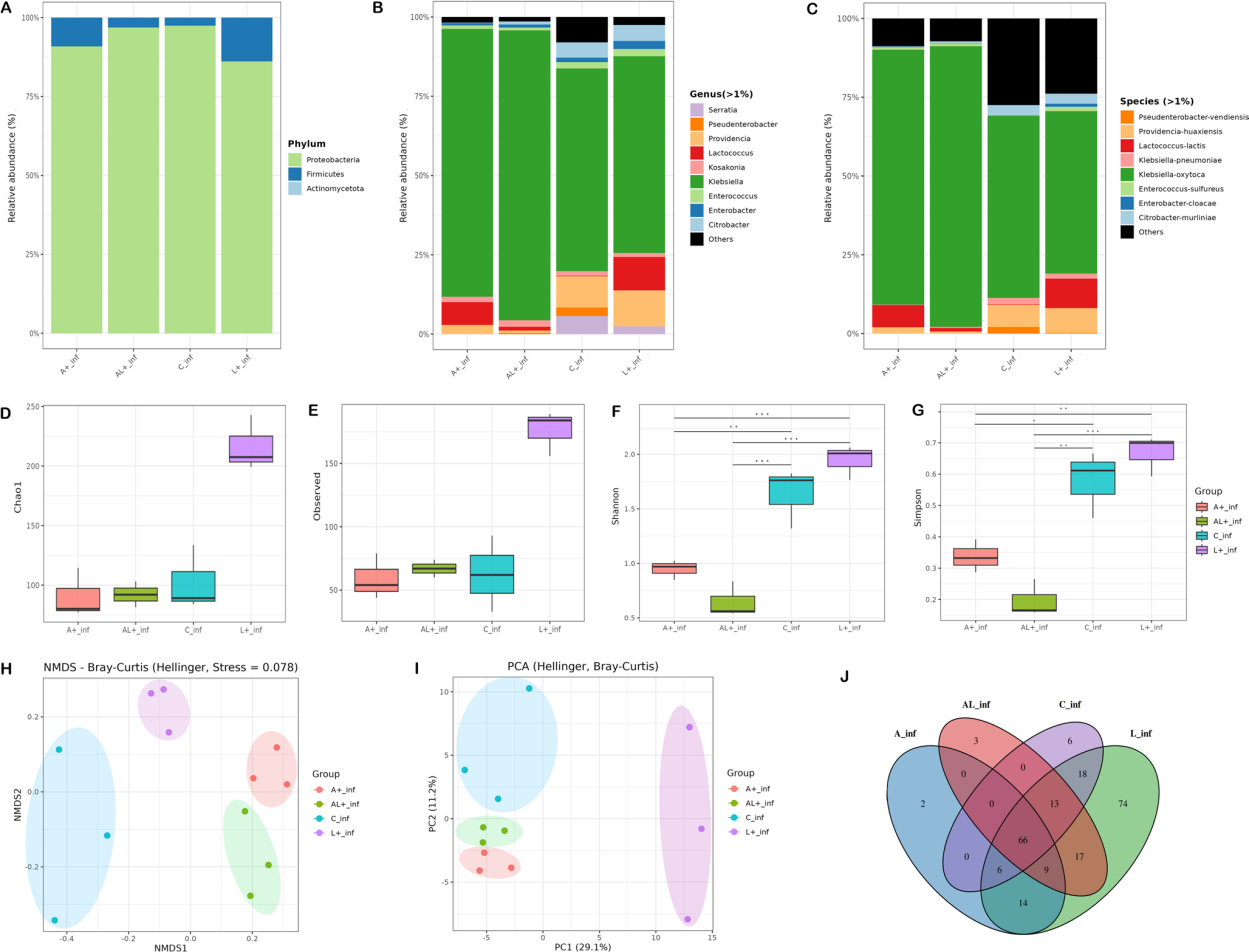
Gut microbiota restructuring in *Ceratitis capitata* following *Pseudomonas* sp. infection depends on prior L.E.K-PC diet. Stacked bars show relative abundances at the phylum (**A**), genus (**B**), and species (**C**) levels. Alpha-diversity indices (**D-G**). Community dissimilarity (Beta-diversity) is visualized by NMDS (**H**) and PCoA (**I**) plots. Panel J is a Venn diagram of OTU distribution. For panel D-G, bars show means ± SD, and (*) denote statistically significant differences (one-way ANOVA, Tukey’s HSD post hoc test, (*P < 0.05, **P < 0.01, ***P < 0.001, ****P < 0.0001))

Following *Pseudomonas* sp. infection, the gut microbiota structure varied across the infected groups (Fig. 4). Although Pseudomonadota remained dominant (> 80%) (Fig. 4A), significant differences in genus and species level composition were detected among treatments (Table S2). All groups were dominated by the genus *Klebsiella* (> 60%), but secondary taxa distinguished each treatment. The L+_inf group showed the highest relative abundances of *Providencia*, *Lactococcus*, and *Citrobacter*, while the C_inf group was enriched in *Providencia*, *Citrobacter*, and *Serratia*. The A+_inf group was characterized by elevated levels of *Lactococcus*, whereas the AL+_inf group exhibited a similar profile but maintained low and stable proportions of non-dominant genera (Fig. 4B).

At the species level, *K. oxytoca* dominated all infected groups. The composition of secondary species, however, varied among groups. *Lactococcus lactis* was most abundant in the L+_inf and A+_inf and *Providencia huaxiensis* was characterized in the C_inf and L+_inf. In contrast, the AL+_inf group contained only low and relatively stable proportions of secondary species, indicating limited diversification beyond the dominant *K. oxytoca* (Fig. 4C) (Table S2).

The patterns of alpha diversity were group-specific. Species richness (Chao1 and Observed OTUs (F _(3, 8)_ = 0.79, *P* < 0.01; F _(3, 8)_ = 0.82, *P* < 0.05, respectively)) was highest in the L+_inf group. In contracts, the A+_inf, AL+_inf, and C_inf groups exhibited lower values, indicating a reduction in bacterial diversity following *Pseudomonas* sp. infection (Fig. 4D and E). The Shannon and Simpson indices (Fig. 4F and G) that account for both richness and evenness also showed significant variation (F _(3, 8)_ = 32, *P* < 0.001; F _(3, 8)_ = 25.73, *P* < 0.001, respectively). In particular, the AL+_inf group displayed the lowest diversity, while the L+_inf and C_inf showed higher values. Beta diversity ordination via NMDS and PCoA (Fig. 4H and I) confirmed distinct compositional differences, with clear separation between all infected groups (PERMANOVA, *P* < 0.001). A Venn diagram illustrated that 68 bacterial taxa constituted a shared core across all treatments post-infection (Fig. 4J).

### Probiotic Conditioning Modulates Microbial Community Shifts in Response to *Pseudomonas* sp. Infection

**Fig. 5:**
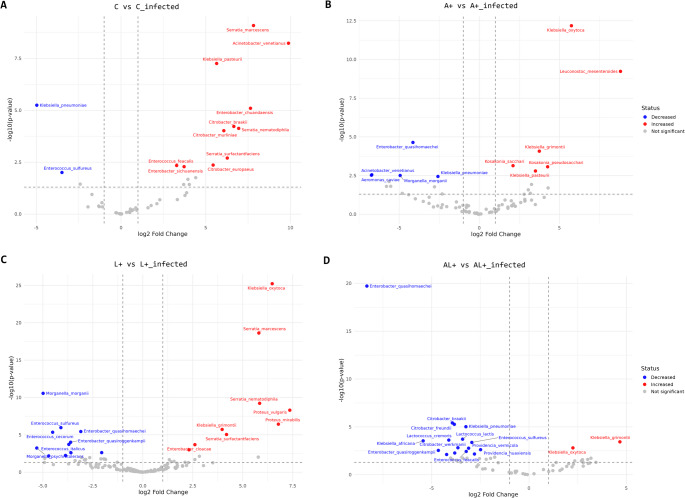
Differential abundance of gut microbiota species in *C. capitata* before and after *Pseudomonas* sp. infection. The volcano plot illustrates the differential abundance of gut microbial species between the dietary groups before (C, A+, L + and AL+) and after infection (C_inf, A+_inf, L+_inf and AL+_inf). Points represent bacterial species. The x-axis represents the log2 fold change, indicating the magnitude of abundance differences, while the y-axis shows the −log10 adjusted P-value, reflecting the statistical significance. Species in red are significantly increased, while species in blue are significantly decreased after *Pseudomonas* sp. infection (adjusted P-value < 0.05). Non-significant species are shown in gray

Differential abundance analysis DESeq2 revealed distinct, diet-dependent shifts in the gut microbiota following *Pseudomonas* sp. infection as visualized in the volcano plot (Fig. 5).

In the control group (C), infection triggered significant changes in 15 species. A notable increase in abundance was observed in 11 taxa, including *Serratia marcesens* (log2 fold change = 7.81; adjusted P-value < 0.0001) and *Acinetobacter venetianus* (log2 fold change = 9.88; adjusted P-value < 0.0001). In contrast, a significant decrease in abundance was recorded for *K. pneumoniae* (log2 fold change=−4.97; adjusted P-value < 0.0001) and *Enterococcus sulfureus* (log2 fold change=−3.48; adjusted P-value < 0.05) after *Pseudomonas* sp. infection (Fig. 5A).

For the A+ group, 6 species exhibited increased abundance after *Pseudomonas* sp. infection, including *K. oxytoca* (log₂ fold change = 5.74, adjusted P-value < 0.0001). In contrast, 4 species showed significant reductions, most notably *Enterobacter quasihormaechei* (log2 fold change = − 4.14, adjusted P-value < 0.001) and *Morganella morganii* (log2 fold change = − 4.93, adjusted P-value < 0.05) (Fig. 5B). The L+ group, showed a balanced response with 9 species increasing, including *K. oxytoca* (log2 fold change = 6.47, *P* < 0.0001) and *Serratia marcescens* (log2 fold change = 5.80, adjusted P-value < 0.0001), and 11 species decreasing. *Morganella morganii* was from the most affected, showing a marked decline (log₂ fold change = − 4.98, adjusted P-value < 0.0001) (Fig. 5C). Finally, the AL+ group showed the most constrained response. Only two species increased in abundance: *K. oxytoca* (log2 fold change = 2.25, adjusted P-value < 0.01) and *K. grimontii* (log₂ fold change = 4.66, adjusted P-value < 0.01). In contrast, 15 species exhibited significant reductions in response to *Pseudomonas* sp. infection reflecting the most pronounced microbial decline among all groups (Fig. 5D).

## Discussion

A balanced gut microbiota plays a pivotal role in nutrient assimilation, metabolic homeostasis, immune activation, and stress tolerance in sterile *C. capitata* males. The emerging concept of immunobiotics, probiotics that enhance host immunity responses via gut microbial modulation, offers a promising strategy to improve the performance of the sterile insect technique (SIT). This study provides a comprehensive experimental evidence that long-term dietary supplementation with the L.E.K-PC consortium enhances the physiological quality, immune responsiveness, and microbial resilience in sterile males challenged with an entomopathogenic bacterium By integrating nutritional assays, immune phenotyping, survival analyses, gene expression profiling, and full-length 16 S rRNA Nanopore sequencing, we establishe a mechanistic link between gut microbiota restoration, metabolic reinforcement, and pathogen resistance.

Our results demonstrate that L.E.K-PC supplementation significantly increased key nutritional reserves, including proteins, carbohydrates, and triacylglycerides, in both larval tissues and adult hemolymph. These metabolic benefits were most pronounced in the AL⁺ group, which received probiotics throughout larval and adult stages, indicating a cumulative effect on nutrient assimilation and energy homeostasis. These findings align with previous studies showing that the restoration or supplementation of beneficial gut symbionts enhances nutrient assimilation and biological performance upon [12, 20, 21, 23, 24, 41–46]. This nutritional enhancement is supported by metagenomic predictions, which indicate an amplification of key microbial functions, including carbohydrate and amino acid metabolism, glycoside hydrolase activity, and cofactor/vitamin biosynthesis [24]. This suggests that the L.E.K-PC consortium improves the host’s nutrient breakdown, absorption, and biosynthetic capacity. Comparable microbiota-dependent nutritional benefits have been reported in other insects. For instance, axenic *Bactrocera dorsalis* larvae exhibit deficits in hemolymph proteins, glucose, trehalose, and triglyceride levels, whichare partially restored after microbial reintroduction [40]. Specific gut bacteria promote larval growth via vitamin B₆ synthesis [47], and dietary probiotics enhance nutrient assimilation and energy storage in *Orius strigicollis* and *Apis mellifera* [48, 49]. Similarly, microbial reintroduction in the red palm weevil restores hemolymph metabolites levels [50], emphasizing the direct contribution of gut symbionts to host nutritional status.

We further demonstrate that L.E.K-PC supplementation enhances immune competence and survival through interconnected nutritional and microbiota-mediated mechanisms. The enrichment of protein reserves provides essential amino acid precursors for synthesising energetically costly immune effectors, including antimicrobial peptides (AMPs), for activating the phenoloxidase (PO) enzymatic cascade in the hemolymph [51]. Consistent with this, probiotic-supplemented males from the AL+ group exhibited significantly higher survival following infection with *Pseudomonas* sp., an entomopathogenic genus associated with gut dysbiosis and an increase in mortality in tephritid flies [11, 22, 27]. This enhanced post-infection survival correlated positively correlated with elevated PO activity and higher antibacterial potency in the hemolymph, indicating a bolstered humoral immune response. Gut symbionts are known to to stimulate immunocompetence by activating the IMD and Toll signaling pathways. Thereby, promoting hemocyte differentiation, and modulating AMP production [32, 52–55]. Thus, the nutritional and metabolic support afforded by L.E.K-PC enrichment ensures the availability of resources required for robust immune system activation in sterile *C. capitata* males following challenge with an entomopathogenic bacterium.

Interestingly, complete immune responsiveness was observed exclusively in the AL⁺_inf group. This group received sustained L.E.K-PC supplementation from the larval stage through adulthood, suggesting that microbial enrichment during critical developmental windows induces more effective state of immune potentiation. This process of immune potentiation has been documented in various insects following exposure to bacterial virulence factors [57, 58, Furthermore gut bacteria are known to modulate systemic melanization through both immune signaling pathways and metabolic co-factors [56]. In line with these findings, our results indicate that early development enrichment with the L.E.K-PC enhances PO activation in sterile *C. capitata* males by providing dual benefits: direct immune stimulation and metabolic support from gut symbionts, which ensures the availability of amino acid precursors required for efficient melanization. Consequently, the enhanced PO activity in the AL⁺_inf group likely explains their increased survival rate following entomopathogen infection. This emphasises that sustained microbial enrichment can not only prime the immune system but also enhance ecological fitness.

The enhanced immune response was characterized by the upregulation of key effector genes such as *Cecropin*,* Attacin*, and the pattern recognition receptor *PGRP*. This expression profile indicates the the concurrent activation of both the Toll and IMD pathways. *Cecropin* and *Attacin* are among the most powerful effectors of humoral immunity in insects, known for their broad efficacy against variouspathogens, including all Gram-negative bacteria such as *Pseudomonas* spp. PGRP serve as a primary sensor of bacterial peptidoglycan and an upstream regulator of the IMD pathway. Elevated PGRP expression in L.E.K-PC enriched males suggests enhanced microbial surveillance and improved capacity of pathogen recognition, facilitating the rapid and targeted deployment of AMPs upon challenge. Probiotic supplementation likely contributes to this effect by modulating gut microbiota composition, thereby promoting a balanced community that supports immune readiness without excessive inflammation [57]. Similar probiotic-driven increases in immune activity have been document across diverse insect taxa. Examples include enhanced increased resistance to entomopathogens in *Tenebrio molitor* [58] and improved immune homeostasis in dipteran models via microbiota-driven potentiation of AMP expression [57, 59]. In tephritid flies, supplementation with beneficial gut bacteria has similarly been linked to increased survival and reduced susceptibility to opportunistic pathogens [24, 60, 61].

At the taxonomic level, L.E.K-PC enrichment established distinct gut microbiota community prior to infection, which strongly influenced subsequent microbial restructuring. This extends our previous Illumina-based findings with the superior resolution of long-read Nanopore sequencing [24]. Pre-infection, all groups were dominated by Pseudomonadota, a core feature of tephritid gut microbiomes known to be essential for nitrogen metabolism, nutrient assimilation, and host fitness [12, 17, 22]. Notably, the AL⁺ treatment uniquely promoted Bacillota, resulting in a more balanced Pseudomonadota-to- Bacillota profile. The species level resolution achieved with Nanopore sequencing revealed clearer associations between specific gut microbiota profiles and functional traits related to immunity and ecological fitness. For instance, the dominance of *K. oxytoca* in the control and A⁺ groups suggest the maintenance of a core, well-adapted gut symbiont. Members of the genus *Klebsiella* are widely recognized for their role in nitrogen recycling, the transformation of nitrogenous compounds, and the enhancement of protein assimilation. These functions provide essential metabolic resources for energetically costly processes like immunity and reproduction [62, 63]. This form of nutritional support likely inderpins the maintenance of key adult performance traits, particularly mating competitiveness, which is crucial for sterile insect technique (SIT) programs [11, 21]. However, supplementation limited to the adult stage appears insufficient to induce genuine developmental immune potentiation, which may explain the relatively reduced capacity to cope with acute infection. In contrast, the predominance of *Enterobacter cloacae* in AL⁺ males highlight the importance of early and prolonged microbial enrichment in the durable structuring of the gut microbiota. Species of the genus *Enterobacter* are frequently associated with (i) Robust stimulation of the IMD and Toll immune pathways, notably through the activation of antimicrobial peptide (AMP) and peptidoglycan recognition protein (PGRP) expression [58, 59, 64], (ii) Nutritional support, including nitrogen recycling and production of metabolites that enhance protein assimilation and energy availability for development and reproduction in *C. capitata* [62, 63]. Colonization established during larval stages, a critical period of intestinal development, promotes priority effects and leads to long-term immune potentiation, enhancing the speed and efficiency of responses against entomopathogenic agents. This combined immunological and nutritional support is consistent with the increased post-infection survival and stress tolerance observed in early-life enriched sterile males, as reported in multiple studies on probiotics and immunobiotics in insects [65, 66]. Finally, the characterization of the L⁺ group by *L. lactis* underscores the specific role of lactic acid bacteria during the larval stage. Through its metabolic activities, including carbohydrate fermentation and the production of organic acids and bioactive metabolites, *L. lactis* enhances nutrient bioavailability, thereby supporting larval growth and metabolic homeostasis that are essential for adult survival and mating competitiveness [23, 61]. In parallel, dietary supplementation with *L. lactis* has been shown to significantly enhance innate immune competence in insects by strengthening multiple key defense mechanisms, including lysozyme activity, the prophenoloxidase cascade (involving prophenoloxidase, serine proteases, and peroxinectin), and expression of immune-related genes, collectively promoting melanization, phagocytosis, and antioxidant defenses [67]. This coordinated activation is associated with increased pathogenic resistance and reduced mortality.

Following entomopathogenic challenge, gut microbiota restructuring was pronounced and treatment-specific, reflecting the combined influence of baseline community composition, probiotic supplementation, and pre-infection diversity. Control-infected flies exhibited enrishement of opportunistic pathogens, including *S. marcescens* and *P. huaxiensis*, taxa known to drive dysbiosis, suppress immunity, and increase mortality [26, 30, 54, 68]. In contrast, L.E.K-PC–supplemented flies maintained beneficial core taxa, notably *K. oxytoca* and *L. lactis*, which promoted competitive exclusion of pathogens and stabilized the microbiota composition. The AL+_inf group, in particular, showed near-monodominance of *K. oxytoca*, reflecting the enhanced microbiota resilience achieved through continuous probiotic exposure. Importantly, the higher pre-infection alpha diversity in supplemented flies contributed to a buffered community response to infection, whereas low-diversity controls experienced severe dysbiosis. These patterns indicate that L.E.K-PC not only enriches beneficial taxa but also enhances overall microbiota diversity, a critical determinant of functional redundancy, ecological stability, and host resistance to pathogens. Similar trends have been reported in other insects. For example, probiotic-supplemented *Bactrocera dorsalis* and *Apis mellifera* maintain stable gut communities, enhanced immunity, and increased survival under pathogen challenge, whereas untreated individuals experience dysbiosis and mortality [40, 47, 49].

Importantly, the application of long-read Nanopore sequencing in this study provided critical advantages over previous short-read approaches. By covering the full V1–V9 hypervariable regions of the 16 S rRNA gene, Nanopore sequencing allowed unambiguous discrimination of closely related species, improving taxonomic resolution to the species level. This high-resolution capability was essential for detecting treatment-specific microbial specific configurations, identifying persistent core taxa under probiotic supplementation, and mapping fine-scale microbiota dynamics following *Pseudomonas* sp. infection. Moreover, full-length sequencing preserves genetic linkage and co-occurrence patterns among taxa, offering insights into potential functional interactions such as cross-feeding for amino acid provisioning, antimicrobial production, and immune potentiation. Long-read sequencing also provides more accurate assessment of α- and β-diversity, allowing us to precisely quantify how probiotic enrichment buffers the microbiota against pathogen-induced collapse. Altogether, these advantages enable a deeper understanding of how L.E.K-PC supplementation shapes the gut microbiota to enhance immune competence, survival, and mating performance. This insight directly informs the optimization of mass-rearing protocols for SIT applications.

## Conclusion

Long-term dietary supplementation with the L.E.K-PC probiotic consortium enhances the physiological, immunological, and microbial fitness of sterile *Ceratitis capitata* males. By promoting a balanced, diverse, and resilient gut microbiota, L.E.K-PC supports nutrient assimilation, immune activation, and pathogen resistance, leading to improved survival and reproductive potential. The application of long-read Nanopore sequencing provided species-level resolution of microbiota dynamics, revealing treatment-specific shifts and functional redundancy critical for host resilience. These findings demonstrate that integrating immunobiotic strategies into SIT programs can optimize male quality, offering a promising approach for enhancing the efficacy of mass-rearing and pest management efforts.

## Supplementary Information

Below is the link to the electronic supplementary material.

Supplementary Material 1 [69] (DOCX 14.0 KB)

Supplementary Material 2 (XLSX 14.2 KB)

## Data Availability

Raw 16 S rRNA reads were deposited in the NCBI Sequence Read Archive (SRA) database under the bioproject number: PRJNA1394647.
