# Supplementary material for: Probiotic Modulation of Gut Microbiota Enhances Immunity and Nutrition in SIT Ceratitis Capitata Sterile Males
Source: Microb Ecol. 2026 Apr 13;89(1):111. doi: 10.1007/s00248-026-02754-x (PMC13186807; doi:10.1007/s00248-026-02754-x)
Supplement: Supplementary file 1 — Supplementary Material 1 [69] (DOCX 14.0 KB) [file 248_2026_2754_MOESM1_ESM.docx]

**Table S1** : Primer pair sequences used to amplify *Ceratitis capitata* immune-related genes

| **Protein product** | **Gene ID** | **Accession** | **Primer pair sequences** | **Reference** |
| --- | --- | --- | --- | --- |
| Cecropin-1 | *Cecropin* | X70030 | F :5’Gcgggttggctgaagaag3’  R : 5’Cggtggctgcgacattag3’ | [69] |
| Attacin A | *Attacin* | FC614 | F : 5’Aaagtgtctacctctcgtttctgg3’  R : 5’Gcatagtagccactcaagtatcgc3’ | [69] |
| PGRP-LC | *PGRP* | HC731 | F : 5’Gcacacaccaaaggctacaatc3’  R : 5’Cacccaaacgaagaccctcatc3’ | [69] |
| G6PDH | *g6pdh* | S67872 | F : 5’ Cggacgagcaggcaaaatatg3’  R : 5’ Agacggacggcggtaagg3’ | [69] |
| GAPDH | *gapdh* | FS831 | F : 5’ Ggtcgcatcggtcgtctgg3’  R : 5’ Gctgaaacggtgcccttgaaac3’ | [69] |
